# Foot type biomechanics in diabetic and not diabetic subjects

**DOI:** 10.1186/1757-1146-5-S1-O13

**Published:** 2012-04-10

**Authors:** Zimi Sawacha, Annamaria Guiotto, Gabriella Guarneri, Angelo aogaro, Claudio Cobelli

**Affiliations:** 1 Department of Information Engineering, University of Padova, Padova, 35100, Italy; 2 Department of Clinical Medicine and Metabolic Disease, University Polyclinic, Padova, 35136, Italy

## Background

The aim of this study was to investigate the role of foot morphology with respect to diabetes and peripheral neuropathy in altering foot kinematics, kinetics and plantar pressure (PP) during gait.

## Materials and methods

Simultaneous 3-dimensional multisegment foot kinematics [1], kinetics and PP [2] of healthy and diabetic subjects with different type of foot were determined. 120 feet were examined (cavus, valgus heel and hallux valgus): 40 feet in the control group (CG), 80 feet respectively in the diabetic ((D) and in the neuropathic (N) groups. Furthermore, subjects were classified according to their foot morphology and each of the 3 groups was splitted in subgroups: 1. cavus foot, 2. cavus foot and valgus heel, 3. cavus foot and hallux valgus, 4. normal foot, 5. cavus foot and normally aligned heel, 6. cavus foot and normal hallux).

## Results

D and N subjects of groups 1, 2 and 5 differed significantly (p<0.05) from CG matched for foot morphology. Most of all D subjects in groups 1 and 2 were significantly more likely to display lower triplanar foot subsegments range of motion (especially in midfoot-forefoot dorsi-plantarflexion angle) and higher peak PP mainly in correspondence of the forefoot.

**Figure 1:**
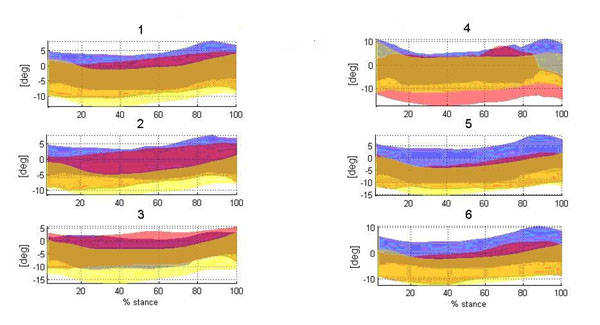
Results for midfoot-hindfoot inversion-eversion angle in each group from 1 to 6 for CG (yellow), D (red), N (blue).

## Conclusions

Results indicated the important role of foot morphology in altering the biomechanics of diabetic subjects.
